# Assessing the Impact of the Leader Peptide in Protease Inhibition by the Microviridin Family of RiPPs

**DOI:** 10.3390/biomedicines12122873

**Published:** 2024-12-18

**Authors:** Jillian L. Stafford, Veronica K. Montoya, Jeffrey J. Bierman, Mark C. Walker

**Affiliations:** Department of Chemistry and Chemical Biology, University of New Mexico, 346 Clark Hall, 300 Terrace St. NE, Albuquerque, NM 87131, USA; jstafford@unm.edu (J.L.S.); vkmontoya@salud.unm.edu (V.K.M.); jeffreybierman@unm.edu (J.J.B.)

**Keywords:** microviridin, elastase, RiPP

## Abstract

**Background:** Ribosomally synthesized and post-translationally modified peptides (RiPPs) are a growing class of natural products biosynthesized from a genetically encoded precursor peptide. RiPPs have attracted attention for the ability to generate and screen libraries of these compounds for useful biological activities. To facilitate this screening, it is useful to be able to do so with the leader peptide still present. We assessed the suitability of the microviridin family for these screening experiments by determining their activity with the leader peptide still present. **Methods:** Modified precursor peptides with the leader present were heterologously expressed in Escherichia coli. Their ability to inhibit elastase was tested with a fluorogenic substrate. HPLC was used to monitor degradation of the modified precursor peptides by elastase. SDS-PAGE was used to determine the ability of immobilized modified precursor peptide to pull down elastase. **Results:** We found that the fully modified precursor peptide of microviridin B can inhibit the serine protease elastase with a low nanomolar IC_50_, and that the fully modified precursor with an N-terminal His-tag can mediate interactions between elastase and Ni-NTA resin, all indicating leader peptide removal is not necessary for microviridins to bind their target proteases. Additionally, we found that a bicyclic variant was able to inhibit elastase with the leader peptide still present, although with a roughly 100-fold higher IC_50_ and being subject to hydrolysis by elastase. **Conclusions:** These results open a pathway to screening libraries of microviridin variants for improved protease inhibition or other characteristics that can serve as, or as inspirations for, new pharmaceuticals.

## 1. Introduction

Compounds produced by living organisms are an important source of pharmaceuticals. Indeed, these natural products and their derivatives account for a third of small molecule drugs in the clinic today [1]. Some of the features that contribute to natural products’ success as drugs, such as their relatively large number of functional groups and stereocenters [2], can also make them challenging to access through organic synthesis. Therefore, there has been a long-standing interest in using the enzymes that biosynthesize these compounds to produce new natural product-like molecules with desired biological activities [3]. Ribosomally synthesized and post-translationally modified peptides (RiPPs) are a large and growing class of natural products that are ideally suited for these efforts [4]. RiPPs are produced from a genetically encoded precursor peptide comprising at least two regions; the core region, which is extensively post-translationally modified, and often a leader peptide, which is recognized by many of the biosynthetic enzymes that install the post-translational modifications [5].

RiPP biosynthesis has garnered attention for the potential to make large libraries of natural product-like compounds that can be screened for new biological activities due to the one-to-one correspondence between the gene encoding the precursor peptide and the final compound, as well as the broad substrate tolerance of many RiPP biosynthetic enzymes. Through screening libraries of the lanthipeptide family of RiPPs, researchers have identified natural product-like compounds that bind streptavidin [6], block the protein–protein interaction between HIV p6 protein and the UEV domain of the human TSG101 protein, which is involved in HIV budding from infected cells [7], as well as lanthipeptides that bind urokinase plasminogen activator [8] and human α_v_β_3_ integrin [9], both of which are involved in cell migration. Libraries of the thiopeptide family of RiPPs have been screened to identify inhibitors of Traf2- and NCK-interacting kinase, which is implicated in a number of cancers [10] as well as thiopeptides that bind the IRAK4 kinase and TLR10 receptor, which are both implicated in inflammation disorders [11]. While much of this work has focused on lanthipeptides and thiopeptides, there are a large number of other families of RiPPs with different post-translational modifications and structures that will be useful to employ in this manner.

**Figure 1:**
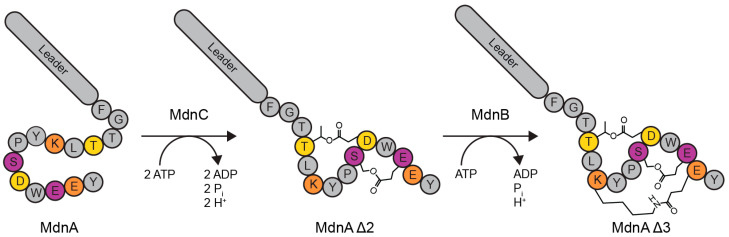
Modification of MdnA. MdnC installs two ester-based macrocycles on the precursor peptide MdnA to produce MdnA Δ2. MdnB then installs one amide-based macrocycle on the precursor peptide MdnA Δ2 to produce MdnA Δ3.

The members of the microviridin family of RiPPs are produced generally by cyanobacteria, although biosynthetic gene clusters encoding their production have been identified in other phyla of bacteria, and are potent inhibitors of serine proteases [12]. These compounds are thought to serve as an antifeedant, as they disrupt molting of *Daphnia* species that feed on cyanobacteria leading to their death [13]. They are post-translationally modified through the installation of ester and amide bonds between serine, threonine, or lysine side chains and aspartate or glutamate side chains, producing a peptide with three macrocycles and may be further modified by the installation of an acetyl group at the N-terminus following proteolytic leader peptide removal [14]. Microviridin B is a representative of this class [15]. The precursor peptide, MdnA, is post-translationally modified by two ATP-grasp ligase family enzymes: MdnC and MdnB. MdnC two ester crosslinks to produce MdnA Δ2 and then MdnB installs an amide crosslink to produce MdnA Δ3 (Figure 1) [16]. In the full biosynthetic pathway, the leader peptide is removed by a protease, and the N-terminus is acetylated to produce Microviridin B, which inhibits the serine protease elastase with low nanomolar affinity [15].

Numerous studies have been performed to alter or enhance the inhibitory activity of microviridins towards proteases [17,18,19]. These efforts have focused on generating constitutively activated modifying enzymes by genetically fusing the leader peptide to the modifying enzyme. These constitutively active modifying enzymes are able to install the post-translational modifications on substrates consisting only of a core peptide. These modified core peptides are then examined for protease inhibition activity without the need for a leader peptide removal step. These studies were able to generate and identify variants of microviridin B and microviridin K, both of which are elastase inhibitors, that were able to inhibit the serine proteases trypsin and subtilisin [17] as well as produce new microviridins identified through genome mining without needing to reconstitute the entire biosynthetic gene cluster [18]. However, these studies have been performed in vitro with a throughput of up to 40 different microviridin variants. To use high throughput techniques that would allow for screening millions or billions of microviridin variants to more thoroughly cover the possible chemical space of microviridin-like compounds, such as phage [20], yeast cell surface [21], or mRNA display [22], it would be beneficial to be able to screen microviridin variants for protease binding while the leader was still present.

Generally, to produce the mature and biologically active RiPP, the leader peptide is removed [23]. While leader peptide removal is common in the biosynthesis of RiPPs, it is not always necessary to generate an active compound. It has been found that the modified precursor peptide of streptolysin S, a RiPP belonging to the linear azol(in)e-containing peptide family, could induce cell lysis, even with the leader peptide present [24]. In another case, the modified precursor peptide of the lanthipeptide haloduracin α was able to inhibit lipid II polymerization in vitro with a similar IC_50_ to the fully mature natural product, although the variant with the leader peptide still present did not display similar levels of antibiotic activity as the mature natural product [25]. The ability to observe biological activity without having to remove the leader peptide decreases the number of manipulations needed prior to screening these compounds. Therefore, we explored the ability of modified MdnA to inhibit elastase.

## 2. Materials and Methods

### 2.1. Materials

All chemicals were purchased from VWR (Solon, OH, USA) unless otherwise noted. Elastase, elastase fluorogenic substrate, and centrifugal concentrator tubes were purchased from Millipore Sigma (Burlington, MA, USA). IPTG and HEPES were purchased from GoldBio (St. Louis, MO, USA). Restriction enzymes, mini-prep kits, gel extraction kits, and NEBuilder were purchased from New England Biolab Corporation (Ipswich, MA, USA). HisPur Ni-NTA resin and agar were purchased from ThermoFisher Scientific (Waltham, MA, USA). Imidazole was purchased from MP BioMedical (Solon, OH, USA). Tris base was purchased from Research Product International Corp. (Prospect, IL, USA). Guanidine hydrochloride was purchased from Macron Fine Chemicals (Stroudsburg, PA, USA). Synthetic double-stranded DNAs (gBlocks) were purchased from Integrated DNA Technologies (Coralville, IA, USA).

### 2.2. Construction of E. coli Plasmids

*E. coli* codon-optimized genes encoding MdnA, MdnB, and MdnC were generated with Integrated DNA Technologies’ codon optimization tool. The plasmid pRSF-mdnA was made by inserting a synthetic double-stranded DNA encoding *E. coli* codon-optimized mdnA into the BamHI and NdeI restriction sites of pRSF-Duet (Millipore Sigma, Burlington, MA, USA). pRSF-Duet was linearized by incubating 3 μg of the plasmid with 50 units of BamHI-HF and 50 units of NdeI in a total volume of 50 μL of 1× CutSmart buffer for 2 h at 37 °C. The linear plasmid was isolated by running the restriction enzyme reaction on a 1% agarose gel and purified with a gel extraction kit. The linear pRSF-Duet and codon-optimized mdnA were assembled by isothermal assembly with NEBuilder as per the manufacturer’s instructions. The plasmid pET-mdnC was made by inserting a synthetic double-stranded DNA encoding *E. coli* codon-optimized mdnC into the BamHI and XhoI restriction sites of pET-Duet (Millipore Sigma, Burlington, MA, USA). pET-Duet was linearized as described for pRSF-MdnA but with BamHI-HF and XhoI, and the linear pET-Duet and codon-optimized mdnC were assembled by isothermal assembly with NEBuilder as per the manufacturer’s instructions. The plasmid pET-mdnB-mdnC was made by inserting a synthetic double-stranded DNA encoding *E. coli* codon-optimized mdnB into the XbaI restriction site of pET-mdnC. pET-MdnC was linearized as described for pRSF-mdnA but with XbaI, and the linear pET-mdnC and codon-optimized mdnB were assembled by isothermal assembly with NEBuilder per the manufacturer’s instructions. All assembled constructs were transformed into chemically competent *E. coli* DH10B (New England Biolab Corporation, Ipswich, MA, USA) following isothermal assembly, and all constructs were confirmed by Sanger sequencing (Azenta Life Sciences, Burlington, MA, USA). Sequences of the synthetic DNA with *E. coli* codon-optimized genes are presented in Supplementary Table S1.

### 2.3. Expression and Purification of MdnA Peptides

The MdnA precursor peptide was produced by transforming *E. coli* BL21(DE3)-T1^R^ (New England Biolab Corporation, Ipswich, MA, USA) with pRSF-mdnA. Two 1 L cultures of terrific broth (TB) supplemented with kanamycin (50 µg/mL) were inoculated to an OD_600_ of 0.05 from an overnight culture of a single colony of freshly transformed *E. coli* BL21(DE3)-T1^R^ and grown at 37 °C with shaking at 200 rpm. When the OD_600_ reached 0.6–0.8, protein expression was induced by adding 1 mM isopropyl β-D-1-thiogalactopyranoside (IPTG), and the cultures were grown with shaking for a further 3 h at 37 °C. The cells were collected by centrifuging at 3000× *g* for 15 min at 4 °C, and the cell paste was stored at −80 °C.

The MdnA Δ2 precursor peptide and MdnA Δ3 precursor peptide were produced by transforming *E. coli* BL21(DE3)-T1^R^ with pRSF-mdnA and pET-mdnC or pET-mdnB-mdnC, respectively. Two 1 L cultures of terrific broth (TB) supplemented with kanamycin (50 µg/mL) and ampicillin (100 µg/mL) were inoculated to an OD_600_ of 0.05 from overnight cultures of single colonies of freshly transformed *E. coli* BL21(DE3)-T1^R^ and grown at 37 °C with shaking at 200 rpm. At an OD_600_ of 0.6–0.8, the cultures were chilled on ice for 30 min; then, 0.5 mM IPTG was added to induce protein expression. The cultures were then grown overnight at 18 °C with shaking. The cells were collected by centrifuging at 3000× *g* for 15 min at 4 °C, and the cell paste was stored at −80 °C.

For purification of all precursor peptides, frozen cell paste was thawed and resuspended in 5 mL per g of cell paste of Buffer A (6M guanidine hydrochloride, 10 mM imidazole, 10 mM Tris base, 100 mM sodium phosphate, 500 mM sodium chloride, pH 8). Cells were lysed by sonication on ice for a total processing time of 3 min with 10 s on and 50 s off. The cell lysate was centrifuged at 20,000× *g* for 30 min at 4 °C to separate soluble and insoluble portions. The remaining purification steps were carried out at room temperature. The clarified cell lysate was passed over a 5 mL HisPur Ni-NTA column by gravity flow. The column was then washed with 20 column volumes Buffer A, followed by 20 column volumes of Buffer A brought up to 20 mM imidazole. The precursor peptides were eluted from the column with 20 column volumes of Buffer A brought up to 500 mM imidazole. Fractions containing the target peptide were identified using UV-Vis spectrometry at 280 nm, pooled, and concentrated in a centrifugal concentrator with a molecular weight cutoff of 3000 Da per the manufacturer’s instructions.

Peptides were further purified by reverse phase chromatography using an Agilent Technologies (Santa Clara, CA, USA) 1260 Infinity II HPLC with Phenomenex (Torrance, CA, USA) Luna 5 µM C18 column, mobile phase of water with 0.2% trifluoroacetic acid (TFA) and acetonitrile (ACN) with 0.2% TFA, 1.0 mL/min flowrate, and a gradient of 2 to 100% ACN over 40 min monitoring at 215 nm and 280 nm. Peaks were manually collected and the fractions containing the peptide of interest were dried by lyophilization. The peptides were resuspended in water prior to their use in assays. The molecular weights of the precursor peptides were verified by mass spectrometry using a Waters (Milford, MA, USA) Xevo G2 XS QToF-MS, and concentration was determined by UV-Vis spectrometry using a calculated ε_280nm_ (ExPASY ProtParam) of 9970 M^−1^ cm^−1^.

To confirm the macrocycle topology of MdnA Δ2 and MdnA Δ3, the peptides were incubated with the protease GluC (0.01 mg of GluC per mg of peptide of interest) in 50 mM ammonium bicarbonate buffer, pH 8, overnight at 37 °C. The samples analyzed on a Thermo Scientific (Waltham, MA, USA) Q-Exactive Orbitrap LC-MS with a Thermo Scientific (Waltham, MA, USA) UltiMate 3000 RSLCnano UHPLC module for liquid chromatography at the Integrative Molecular Analysis Core at the University of New Mexico to obtain tandem mass spectrometry data.

### 2.4. Elastase Inhibition Assay

Elastase inhibition was determined using a modified literature method [26] to measure the hydrolysis of the model peptide substrate MeOSuc-AAPV-AMC. Assays were performed at room temperature in a total volume of 100 µL containing 100 mM HEPES, pH 8.0, 10% DMSO, 20 nM elastase, and 0.1 nM–100 nM MdnA Δ3 or 10 nM–10,000 nM MdnA Δ2. The reaction was initiated by adding the fluorogenic substrate (160 µM). Elastase and inhibitor peptide were added to the well in a total volume of 50 µL with 100 mM HEPES and incubated at room temperature for 10 min before additional HEPES, DMSO, and substrate were introduced. Fluorescence was measured on a BioTek (Winooski, VT, USA) Synergy H1 microplate reader equipped with a 360/40 excitation filter and a 460/40 emission filter in a black 96-well plate, measuring fluorescence every 10 s for 10 min. The rate of hydrolysis was determined using linear regression in Microsoft (Redmond, WA, USA) Excel. IC_50_s were determined by fitting to the Dose Response Curve in Origin, OriginLab (Northampton, MA, USA).

### 2.5. MdnA Hydrolysis Assay

Assays to determine MdnA Δ2 and MdnA Δ3 susceptibility to hydrolysis by elastase were performed at room temperature in a total volume of 100 µL containing 100 mM HEPES, pH 7.5, 3.9 µM elastase, and 70 µM of MdnA Δ2 or MdnA Δ3. Reactions was incubated at room temperature for 10, 35, and 60 min. The reactions were halted by injection on an Agilent Technologies (Santa Clara, CA, USA) 1260 Infinity II HPLC with 150 × 2.1 mm Phenomenex Kinetex 5 µM C18 column with water with 0.2% TFA and ACN with 0.2% TFA mobile phase, 1 mL/min flowrate, 50 °C column compartment, and gradient of 20–50% ACN over 10 min, with monitoring at 215 nm and 280 nm. All reactions were performed in triplicate. Peaks in the chromatogram were integrated in OpenLab CDS, Agilent Technologies (Santa Clara, CA, USA), and compared to MdnA Δ2 and MdnA Δ3 standards with known concentration.

### 2.6. Elastase Pulldown Assay

Reactions with MdnA Δ3 (80 µM), elastase (8 µM), and MdnA Δ3 and elastase were incubated in HEPES buffer (100 mM, pH 7.5) in a total volume of 500 µL for 10 min at room temperature. The reactions were then chilled on ice, added to 50 µL of Ni-NTA resin, and incubated for 1 h with occasional agitation. The Ni-NTA resin was pelleted by centrifugation at 500× *g* for 1 min. The supernatant was removed by pipetting, and the Ni-NTA resin was washed twice with 1 mL of Buffer B (8 M urea, 100 mM sodium phosphate, 10 mM Tris, 10 mM imidazole, pH 8) with centrifuging to pellet the Ni-NTA resin after each wash. Material bound to the Ni-NTA resin was eluted with Buffer B brought to 500 mM imidazole. A total of 18 μL of the eluent was added to 6 μL of SDS-PAGE loading buffer and incubated at 95 °C for 10 min. These samples were then run on a 4–20% Tris/glycine SDS-PAGE gel (Bio-Rad, Hercules, CA, USA) and the bands visualized by staining with Coomassie brilliant blue.

## 3. Results

### 3.1. Elastase Inhibition

To determine whether the tricyclic MdnA Δ3 could inhibit elastase, MdnA with an N-terminal His-tag was coexpressed with MdnB and MdnC in *E. coli* and purified by immobilized metal affinity chromatography (IMAC). Mass spectrometry of the purified MdnA Δ3 revealed the expected 3-fold dehydration from the formation of two lactone and one lactam macrocycles (Supplementary Table S1). The formation of the expected macrocyclization topology was examined using tandem mass spectrometry following digestion by the protease Glu-C to isolate the core region of MdnA Δ3. Limited fragmentation was observed within the macrocyclized portion of the core region, suggesting the expected macrocycles were installed (Supplementary Figure S1 and Supplementary Table S2).

**Figure 2:**
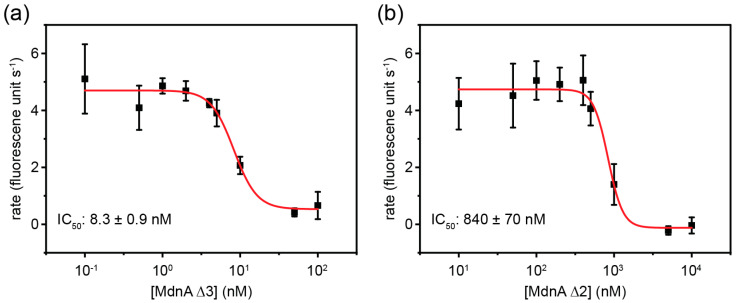
Inhibition of elastase by MdnA Δ3 and MdnA Δ2. Inhibition of the hydrolysis of a model fluorogenic substrate by MdnA Δ3 (**a**) and MdnA Δ2 (**b**). Rate measurements were performed in triplicate. Error bars represent the standard deviation. The red line represents the line of best fit.

The ability of MdnA Δ3 to inhibit the hydrolysis of a model peptide substrate, MeOSuc-AAPV-AMC, by elastase was then examined. MdnA Δ3 was found to inhibit the reaction with an IC_50_ of 8.3 ± 0.9 nM (Figure 2).

Having seen MdnA Δ3 was able to inhibit elastase, the ability of bicyclic MdnA, called MdnA Δ2, that had only been modified by MdnC was explored. To access MdnA Δ2, MdnA with an N-terminal His-tag was coexpressed with MdnC in *E. coli* and purified by IMAC chromatography. Again, mass spectrometry revealed the expected 2-fold dehydration from the formation of two lactones (Supplementary Table S2), and tandem mass spectrometry produced fragments consistent with the installation of the expected macrocycle topology (Supplementary Figure S2). MdnA Δ2 was found to inhibit the hydrolysis of MeOSuc-AAPV-AMC by elastase with an IC_50_ of 840 ± 70 nM (Figure 2).

### 3.2. Elastase Activity on Precursor Peptides

To determine if elastase is catalyzing the hydrolysis of linear portions of MdnA Δ3 or MdnA Δ2, these peptides were incubated with elastase and quantified by HPLC.

**Figure 3:**
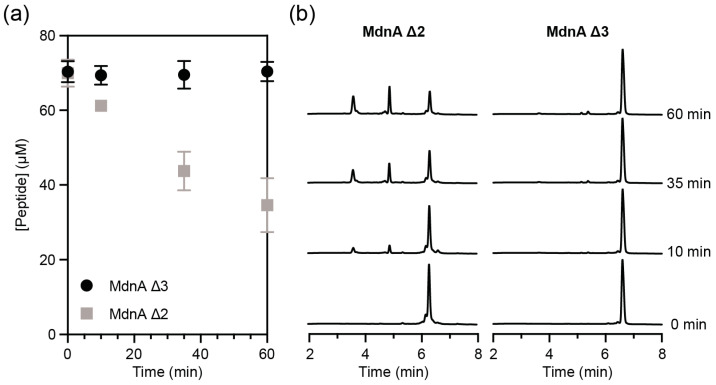
Degradation of MdnA by elastase. (**a**) Quantification of MdnA Δ3 and MdnA Δ2 remaining following treatment with elastase. Measurements were performed in triplicate, and error bars represent standard deviation. (**b**) Chromatograms demonstrating hydrolysis of MdnA Δ2 and the lack of hydrolysis of MdnA Δ3. New peaks appearing in the MdnA Δ2 chromatograms represent hydrolysis products of MdnA Δ2. All chromatograms are shown at the same scale.

Over time, MdnA Δ2 was consumed by elastase, while the concentration of MdnA Δ3 remained relatively constant over the time the reaction was monitored (Figure 3). The pseudo-first order rate constant at which MdnA Δ2 is hydrolyzed by elastase over the first 10 min is 3.8 × 10^−3^ ± 7.2 × 10^−4^ s^−1^.

### 3.3. Elastase Binding MdnA Δ3

**Figure 4:**
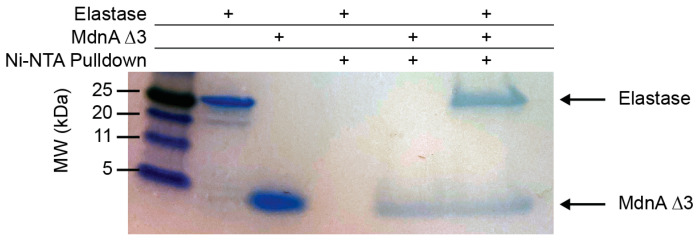
Elastase pulldown. Elastase and MdnA Δ3 lanes are presented as standards. MdnA Δ3 and elastase in the presence of MdnA Δ3 can be pulled down using Ni-NTA resin, but elastase on its own cannot be. The + indicates the presence of the respective components in each sample.

Given MdnA Δ3 inhibits elastase and is not subject to hydrolysis by it, we sought to demonstrate MdnA Δ3 can directly bind elastase. To do so, we used His-tagged MdnA Δ3 to mediate an interaction between Ni-NTA resin and elastase. His-tagged MdnA Δ3 was incubated with elastase, and the mixture was incubated with Ni-NTA resin. Following incubation, the resin was washed, and the His-tagged MdnA Δ3 was eluted from the resin. The eluent from the resin contained elastase only when MdnA Δ3 was also present (Figure 4 and Supplementary Figure S3).

## 4. Discussion

We found that MdnA Δ3 is able to reduce the rate of hydrolysis of a model substrate MeOSuc-AAPV-AMC by elastase in a dose-dependent manner. Although, the IC_50_ value we measured is certainly an overestimation of the actual IC_50_ as it is less than half the concentration of the elastase used in the assay. This value is nonetheless the same order of magnitude as the previously reported IC_50_ for elastase inhibition by microviridin B, 25 nM [8], despite the presence of the leader peptide in MdnA Δ3. This result suggests that the presence of the leader peptide does not interfere with elastase inhibition. We also found that MdnA Δ2 is able to reduce the activity of elastase in a dose-dependent manner, although with a higher IC_50_ compared to that of MdnA Δ3. It has previously been observed that other microviridins, in the absence of their leader peptides, exhibit a similar 100-fold decrease in their inhibitory activity when they are bicyclic rather than in their native tricyclic form [17], suggesting this increase in the IC_50_ is due to the lack of the third macrocycle rather than the presence of the leader peptide. The lack of the third macrocycle may allow MdnA Δ2 to adopt non-binding conformations, which are inaccessible to MdnA Δ3 due to the structural restraint imposed by a third macrocycle, thus reducing the affinity of MdnA Δ2 for elastase.

As elastase cleaves amide bonds after small hydrophobic amino acid residues [27], we hypothesized that it was possible that elastase is binding to the leader peptide of MdnA Δ3 or MdnA Δ2, which contain such residues, and catalyzing the hydrolysis of the amide backbone. If this situation were the case, the reduction in rates of hydrolysis of the model substrate by elastase observed above could be due to MdnA Δ3 or MdnA Δ2 competing as a substrate rather than inhibiting elastase. When elastase is incubated with MdnA Δ3 no degradation of the peptide is observed within the limit of detection, suggesting that the reduction in the rate of hydrolysis of the model substrate is due to inhibition of elastase and not MdnA Δ3 serving as a competitive substrate. Additionally, when elastase is incubated with MdnA Δ2, degradation is observed; however, the rate at which MdnA Δ2 is consumed by elastase is approximately three orders of magnitude less than the reported k_cat_ of elastase for MeOSuc-AAPV-AMC, 16.8 s^−1^ [28]. This low apparent rate of hydrolysis of MdnA Δ2 by elastase suggests that the reduction in the rate of hydrolysis of the model substrate is largely, but not entirely, due to MdnA Δ2 acting as an inhibitor. The degradation of MdnA Δ2 that is observed is likely due to some fraction of elastase not being inhibited by binding the core portion of MdnA Δ2 and therefore being able to bind to the linear leader peptide and catalyze the hydrolysis of the amide backbone.

Given low nanomolar IC_50_ we observed for MdnA Δ3 inhibiting elastase, we hypothesized that it was possible that elastase cleaved the leader peptide from a small portion of MdnA Δ3 and that this species, without a leader peptide, was responsible for inhibiting elastase, and thereby preventing further consumption of MdnA Δ3. Were this the case, elastase would be bound to this truncated peptide lacking a His-tag rather than MdnA Δ3 with a His-tag. Our pulldown assay demonstrates that while elastase on its own does not bind Ni-NTA resin, MdnA Δ3 is able to mediate an interaction between the Ni-NTA resin and elastase. This result demonstrates that elastase is interacting with MdnA Δ3 that retains its His-tag, which therefore must be interacting with MdnA Δ3 with the leader peptide still present.

The interaction between MdnA Δ3 and elastase opens the potential to use surface display platforms that were developed for screening libraries of proteins, such as yeast surface display or phage display, to screen libraries of MdnA variants for improved elastase inhibition activity or for other parameters such as improved stability or solubility. Uncontrolled elastase activity has been implicated in diseases such as pulmonary emphysema and rheumatoid arthritis; therefore, identifying natural product-like inhibitors could help with treatment [29]. These MdnA variant libraries could also be screened for the ability to inhibit other serine proteases, which have been implicated in a large number of diseases [30]. Development of this platform would enable the identification of new natural product-like compounds that could serve as, or as inspirations for, new pharmaceuticals.

## 5. Conclusions

The ability of MdnA Δ3 to inhibit the activity of elastase and mediate an interaction between elastase and Ni-NTA resin demonstrate that removal of the leader peptide is not necessary for serine protease binding and inhibition by modified microviridin precursor peptides, although the full three macrocycles are necessary to achieve the tightest binding. It remains unknown whether leader peptide removal is essential for microviridins to carry out their biological activity in vivo, such as inhibiting molting in *Daphnia*. These results suggest microviridins are a suitable platform for generating libraries of natural product-like compounds that can be screened for new and useful biological activities.

## Data Availability

Raw data are available from the corresponding author.

## Supplementary Materials

The following supporting information can be downloaded at: https://www.mdpi.com/article/10.3390/biomedicines12122873/s1, Table S1: Synthetic double-stranded DNA used in this study; Figure S1: Tandem mass spectrometry of MdnA Δ3; Figure S2: Tandem mass spectrometry of MdnA Δ2; Table S2: Study Mass spectrometry of MdnA precursor peptides. Figure S3: Original gel used for Figure 4.
