# Supplementary material for: Assessing the Impact of the Leader Peptide in Protease Inhibition by the Microviridin Family of RiPPs
Source: Biomedicines. 2024 Dec 18;12(12):2873. doi: 10.3390/biomedicines12122873 (PMC11672978; doi:10.3390/biomedicines12122873)

Supplementary Information For

Leader peptide removal is not necessary for elastase inhibition by microviridin B

Jillian L. Stafford,[a] Veronica K. Montoya,[a] Jeffrey J. Bierman,[a] and Mark C. Walker*[a]

Supplementary Table S1. Synthetic double stranded DNA used in this study.

Name	Sequence
**mdnA**	CATCACCACAGCCAGATGGCCTACCCCAACGACCAGCAAGGCAAAGCACTTCCTTTTTTTGCTCGTT<br>TCCTGTCAGTCTCCAAGGAGGAGTCGTCTATTAAATCACCCAGCCCAGAACCTACATTTGGCACTAC<br>GTTAAAGTACCCTTCGGACTGGGAAGAGTACTGAGAGTCTGGTAAAGAAACC
**mdnC**	GTTTAACTTTAAGAAGGAGATATACCATGACTGTTCTGATTGTGACGTTTAGTCACGATAATGAGTCT<br>ATCCCTCTGGTGATCAAAGCGATCGAGACGATGGGCAAAAAAGCGTTCCGCTTTGATACTGATCGC<br>TTTCCCACGGAGGTCAAGGTAGATCTGTATAGCGGTGGGCAAAAAGGAGGCATTATCACAGACGGC<br>GACCAGAAACTTGAGCTGAAGGAAGTTTCAGCCGTCTGGTATCGTCGCATGCGTTACGGACTGAAG<br>CTGCCAGATGGTATGGACTCGCAGTTCCGCGAAGCTTCACTGAAAGAGTGTCGTTTGAGCATCCGT<br>GGCATGATTGCCTCTTTGAGTGGGTTTCATCTTGACCCCATTGCAAAAGTAGATCACGCCAACCACA<br>AACAGTTGCAACTGCAGGTGGCGCAGCAGCTTGGATTGCTGATCCCAGGGACTTTGACCTCAAACA<br>ATCCTGAAGCTGTGAAACAATTTGCCCAGGAATTTGAAGCGACTGGCATCGTTACTAAGATGCTTAG<br>CCAGTTCGCCATTTATGGTGATAAGCAGGAGGAGATGGTGGTTTTTACCTCCCCGGTCACTAAGGA<br>AGATCTGGACAACTTGGAAGGTTTACAGTTTTGCCCTATGACCTTCCAAGAGAACATTCCAAAGGCT<br>CTGGAGTTACGTATCACGATCGTAGGAGAACAGATTTTCACGGCAGCGATCAACTCGCAACAGCTT<br>GATGGGGCCATTTACGATTGGCGTAAAGAGGGTCGCGCTCTGCACCAACAATGGCAACCATACGAC<br>CTTCCCAAAACCATTGAGAAACAGTTACTGGAGTTAGTTAAGTATTTTGGGTTAAACTATGGAGCAAT<br>CGATATGATCGTCACACCAGATGAACGCTATATTTTCTTGGAGATTAACCCCGTGGGCGAATTTTTCT<br>GGTTGGAGTTGTATCCTCCTTATTTTCCGATTAGCCAGGCAATCGCGGAGGTCCTGGTGAACTCCTG<br>AGAGTCTGGTAAAGAAACC
**mdnB**	GCGGATAACAATTCCCCTCTAGAAACGTCGACTTATCTCGAGACTGCAGTTCAATAGAGATATTGTT<br>GACGGTACCGTATTTTCGTATTTTGGATGACTAGAGAAAGAGGAGAAATACTAGATGAAAGAATCAC<br>CAAAGTAGTTTTACTTTTAACACATAGCGGTGACTTCTTTACAATCGACCGCGTCGCAGAGGCTATC<br>GAGAAAAAAGGCGCGACGCCGTTCCGTTTGGATACCGATAAATTCCCACTGGAAGTGCAATTAGCC<br>GCTCAGTTTAATGGCAAAAAGTCTTTTTACCAGCTTACCTATAACCACCAGTCAATTGATTCAGAACA<br>AGTGCAATCCGTATGGACGCGCCGTATCTGGCAGCCGGAGATCACGGGCGATTTAGATCCTCAATT<br>TCGCGAAGCATGCGTTCGCGAGTCTCAGACAACTCTTGCGGGGTTTTGGGATTCACTTCGTTCGGC<br>TCGTTGGTTGGACAATTTAGCGCAAATTGAGCGTGCGAAGAATAAACTGCTGCAGCTGCGCCTTGC<br>ATCAGAGGTCGGACTTATTATTCCCCCTACACTTGTTACGAACAATCCTGATGCCGCTCGCGAGTTC<br>TTCAGCCAGGTTCAAGGGCGCATGGTCTCGAAGCTGTTGACTGCCATTGCCCGTTCTATGGAGTCG<br>CCTGAATTTTTTTTATACACATCCCGCGTTAAGGCTGAGGATTTGGAAGAAGCGGAATCCCTGCGTT<br>ATTGCCCAATGGTGTTTCAAGCTGAGATTCCGAAGCAACTTGAATTGCGTGTCGTCGTAGTAAATGG<br>TCAAACTTTTGTCGGCGCCTTAGAGTCTAGCCAATACAATAATTCGGCAGTCGATTGGCGTCGCCCA<br>GGCATCGACCCTGGGGCATGGCAGCATCACACACTGCCAGATTCCCTTTTGCAGCAGTTACAGATC<br>TTCATGGCGAACCTGGGCCTTAATTTCGGGGCTTTTGATTTTATCCTTACTCCCGGTGGTGAGTATG<br>TGTTCCTTGAGGTCAATCCTGGCGGTGAATGGGGAATGCTTGAACGTGATTTGGATTTACCCATCTC<br>AAACGCTATTGCTGACTTCTTGGTGTGTTGAAAGAAGGAGATATACCATGACTGTTCTGATTGTGAC<br>G

Supplementary Table S2.

Peptide	Charge State	Expected Mass (m/z)	Observed Mass (m/z)	Δ ppm
MdnA	+8	872.0416	872.0048	42
MdnA Δ2	+8	867.5383	867.5020	42
MdnA Δ3	+8	865.2876	865.2504	43

Supplementary Figure S1. Tandem mass spectrometry of MdnA Δ3.

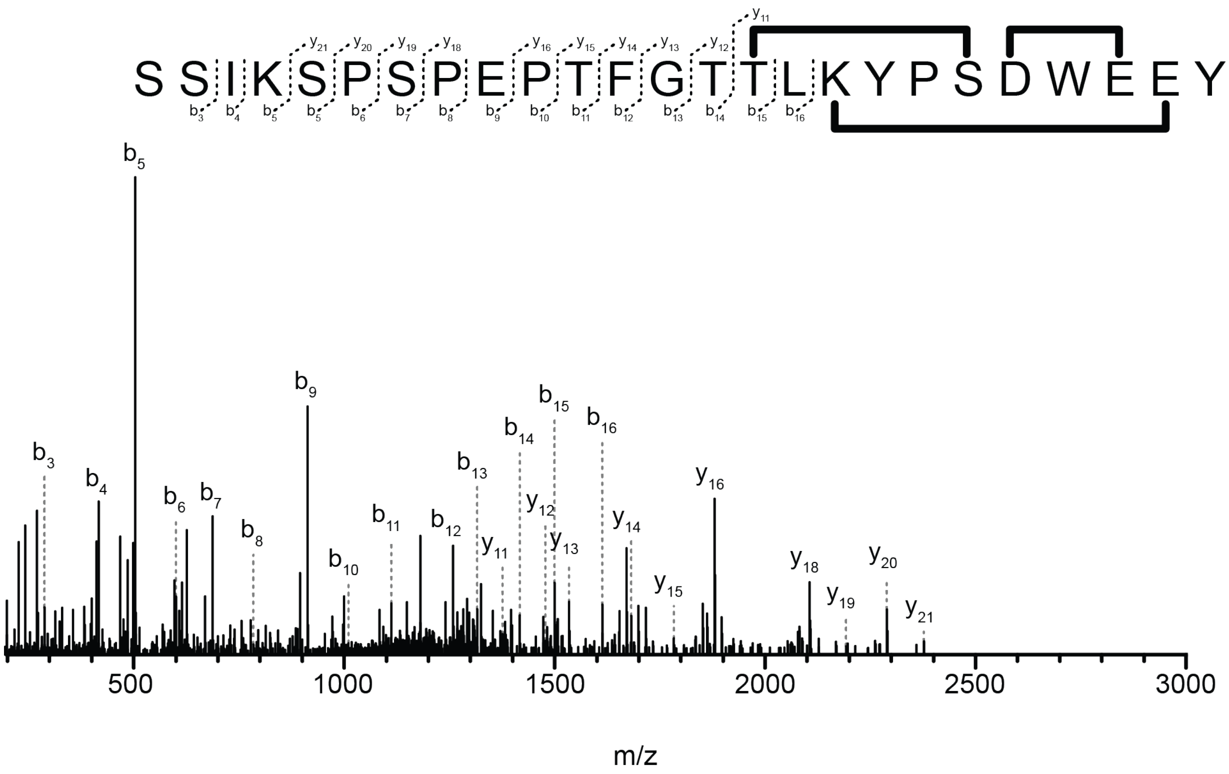

Ion	Predicted (m/z)	Observed (m/z)	Δ PPM
b_3	288.1554	288.1543	4
b_4	416.2504	416.2488	4
b_5	503.2824	503.2806	4
b_6	600.3352	600.3336	3
b_7	687.3672	687.3648	3
b_8	784.4199	784.4176	3
b_9	913.4625	913.4590	4
b_{10}	1010.5153	1010.5061	9
b_{11}	1111.5630	1111.5552	7
b_{12}	1258.6314	1258.6281	3
b_{13}	1315.6529	1315.6423	8
b_{14}	1416.7005	1416.6986	1
b_{15}	1499.7326	1499.7372	3
b_{16}	1612.8167	1612.8179	1
y_{11}	1376.6006	1376.6168	12
y_{12}	1477.6483	1477.6560	5
y_{13}	1534.6698	1534.6788	6
y_{14}	1681.7382	1681.7482	6
y_{15}	1782.7859	1782.8140	16
y_{16}	1879.8386	1879.8442	3
y_{17}	2105.9340	2105.9368	1
y_{19}	2192.9660	2192.9597	3
y_{20}	2290.0188	2290.0251	3
y_{21}	2377.0508	2377.0574	3

Supplementary Figure S2. Tandem mass spectrometry of MdnA Δ2.

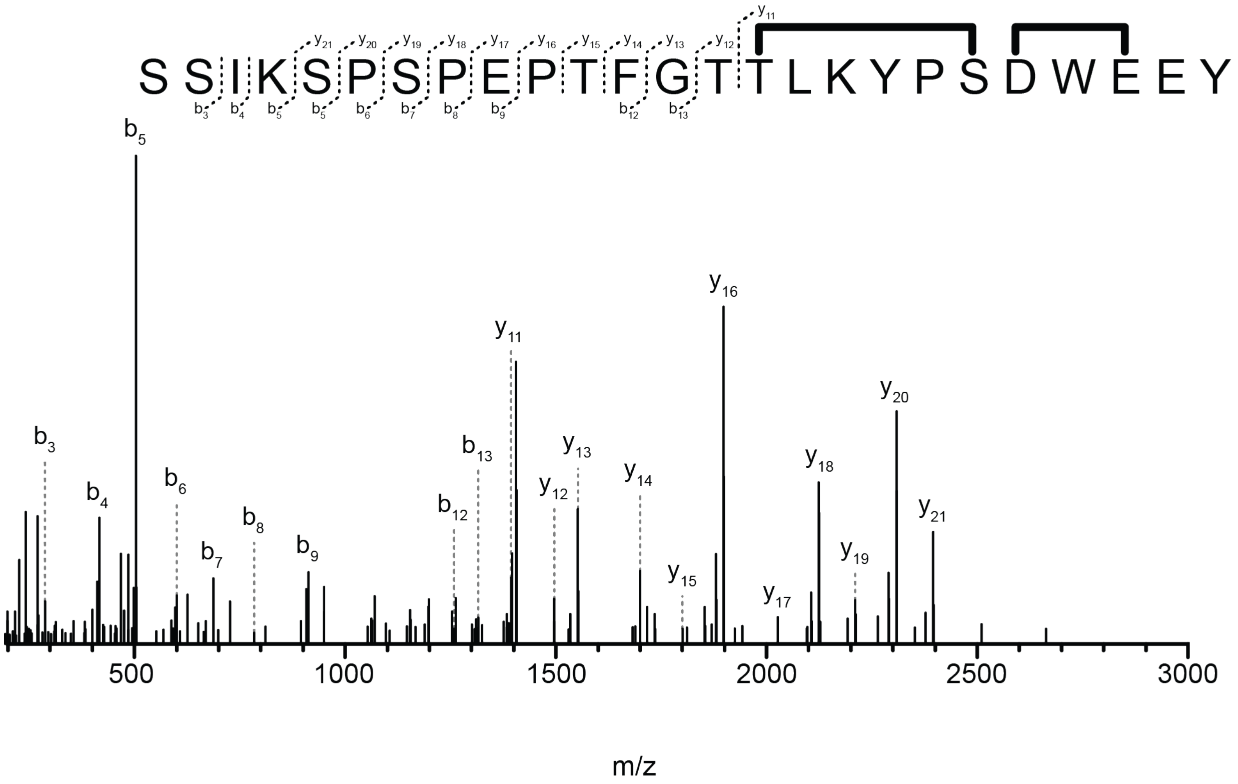

Ion	Predicted (m/z)	Observed (m/z)	Δ PPM
b_3	288.1554	288.1532	8
b_4	416.2504	416.2480	6
b_5	503.2824	503.2794	6
b_6	600.3352	600.3304	8
b_7	687.3672	687.3636	5
b_8	784.4199	784.4213	2
b_9	913.4625	913.4571	6
b_{12}	1258.6314	1258.6224	7
b_{13}	1315.6529	1315.6332	15
y_{11}	1394.6162	1394.6232	5
y_{12}	1495.6639	1495.6643	0.3
y_{13}	1552.6854	1552.6855	0.1
y_{14}	1699.7538	1699.7546	0.5
y_{15}	1800.8015	1800.7810	11
y_{16}	1897.8542	1897.8530	0.6
y_{17}	2026.8968	2026.8810	8
y_{18}	2123.9496	2123.9451	2
y_{19}	2210.9816	2210.9795	0.9
y_{20}	2308.0344	2308.0286	3
y_{21}	2395.0664	2395.0601	3

Supplementary Figure S3. Original gel for Figure 4.

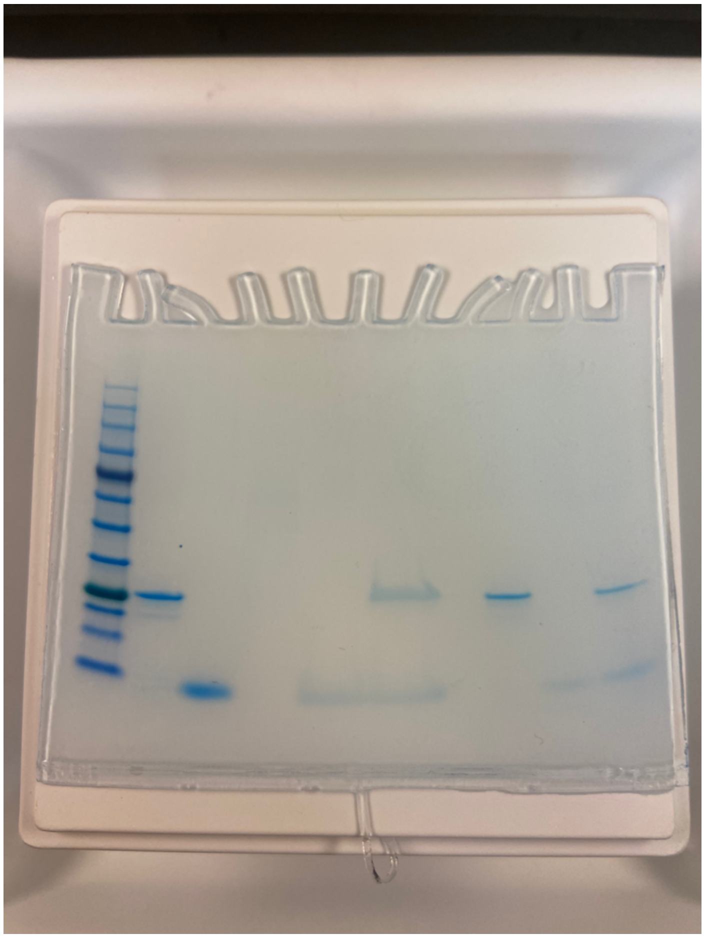

Supplement: Supplementary file 1 [file biomedicines-12-02873-s001.zip › biomedicines-3335923-supplementary.pdf]
